# Nonsteroidal antiinflammatory drugs alter antibiotic susceptibility and expression of virulence-related genes and protein A of Staphylococcus aureus

**DOI:** 10.3906/sag-2003-60

**Published:** 2021-04-30

**Authors:** İsmail ÖZTÜRK, Yasemin ERAÇ, Petek BALLAR KIRMIZIBAYRAK, Şafak ERMERTCAN

**Affiliations:** 1 Department of Pharmaceutical Microbiology, Faculty of Pharmacy, İzmir Katip Çelebi University, İzmir Turkey; 2 Department of Pharmacology, Faculty of Pharmacy, Ege University, İzmir Turkey; 3 Department of Biochemistry, Faculty of Pharmacy, Ege University, İzmir Turkey; 4 Department of Pharmaceutical Microbiology, Faculty of Pharmacy, Ege University, İzmir Turkey

**Keywords:** Gene expression, nonsteroidal antiinflammatory drugs, *Staphylococcus aureus*, antibiotic sensitivity, real-time qRT-PCR, immunoblotting

## Abstract

**Background/aim:**

Nonsteroidal antiinflammatory drugs (NSAIDs) including diclofenac, naproxen, ibuprofen, acetylsalicylic acid, and acetaminophen have been shown to have antimicrobial effects on various microorganisms. The aim of this study was to investigate the antibacterial effects of NSAIDs on *Staphylococcus aureus* .

**Materials and methods:**

Susceptibilities of *S. aureus* strains to NSAIDs with or without antimicrobials (moxifloxacin, vancomycin, ciprofloxacin, clindamycin, and gentamicin) were determined using the microdilution method and disk diffusion test. Expression levels of genes in the presence of drugs were investigated by real-time quantitative RT-PCR (qRT-PCR), and immunoblotting analysis was performed for staphylococcal protein A (SpA).

**Results:**

Our results showed that all NSAIDs were active against *S. aureus* strains with MIC values ranging from 195 µg/mL to 6250 µg/mL. NSAIDs increased the antibiotic susceptibility of the strains, and diclofenac was found to be more effective than the other drugs. Drugs showed different effects on expression levels of virulence factor and/or regulatory genes. Immunoblotting analysis of SpA protein was mostly in accordance with qRT-PCR results.

**Conclusion:**

The regulatory/virulence factor genes and proteins of *S. aureus* investigated in this study may be reasonable targets for these drugs, and we suggest that the data may contribute to the field of infection control and antimicrobial resistance.

## 1. Introduction

Methicillin-resistant *Staphylococcus aureus* (MRSA) clones were first detected in European hospitals during the 1960s. MRSA rapidly spread across international borders, and is now increasingly recognized in the community [1,2]. MRSA has emerged as a cause of various community-associated infections in both pediatric and adult populations from rural and urban areas [1,3]. MRSA strains have become resistant to many beta-lactam agents, macrolides, aminoglycosides, and lincosamides [1,4]. As a result of resistance, vancomycin usage has increased, and more recently, emergence of vancomycin-intermediate and vancomycin-resistant *S. aureus* strains have become problematic [5]. In recent decades, MRSA has acquired a dramatic relevance in human medicine for different reasons and still continues to be a major cause of health care-related and community-associated infections with multidrug-resistant strains [6,7].

*Staphylococcus aureus* has diverse components and products that contribute to the pathogenesis of infection, and these factors can act together or alone [8]. *Staphylococcus aureus* produces a variety of pyrogenic toxins and super-antigens, and forms biofilms on tissues and medical devices, which cause drug resistance and difficulty in treatment [4,6]. It is not possible to describe the vast majority of *S. aureus* infections with the effect of a single virulence component, indeed it is likely that a number of virulence factors, including toxins, cell wall-associated adhesins, and secreted exoproteins, can act in combination in the process of diseases [9]. The accessory gene regulator (agr), staphylococcal accessory regulator ( *sar* ), multidrug efflux pump gene repressor ( *mgr* ) and sigma factor B ( *sigB* ) have been identified as regulatory genes in *S. aureus* coordinating the expression of various groups of virulence factors. Staphylococcal protein A (SpA), a 42 kDa surface protein, represents an important virulence factor that has the ability to interact with immune system components including immunoglobulin (Ig) molecules. SpA is covalently anchored to the cell wall of most *S. aureus* strains and contains highly homologous extracellular Ig-binding domains in tandem [10–12]. Multidrug efflux is one of the critical mechanisms responsible for resistance in *S. aureus* via removal of drug from its site of action. Multidrug and toxin extrusion (MATE) family multidrug efflux pump (MepA) is a member of multidrug resistance-conferring efflux pumps that are based on structural characteristics and energy requirements [13]. Sortase (SrtA), a membrane protein in *S. aureus* , is a thiol transpeptidase that tethers cell surface components to the cell wall [14,15]. Expression of virulence genes may have clinical importance, and different stages of infections appear to require different expression patterns of virulence determinants [1,16]. Antibiotics, antiinflammatory, analgesic, and antipyretic drugs are used in the treatment of bacterial infections and inflammation, alone or in combination. The term “nonantibiotic” was coined by Kristiansen [17] for certain drugs that have a greater/lesser degree of broad-spectrum antibacterial activity, and most studies have focused on nonsteroidal antiinflammatory drugs (NSAIDs) [18].

The aim of this study was to investigate the antibacterial effects of frequently used antiinflammatory, analgesic, and antipyretic drugs on clinical and nonclinical *S. aureus* strains. For this purpose, antibacterial effects of diclofenac, naproxen, ibuprofen, acetylsalicylic acid, and acetaminophen were evaluated using phenotypic methods. The effects of these drugs on the expression of genes ( *sarA* , *agr* RNAIII, *sigB* , *mgrA* , *spa* , *mepA,* and *srtA* ) and SpA were also investigated using transcriptional and protein experiments.

## 2. Materials and methods

### 2.1. Bacterial strains and chemicals

The present study utilized three clinical MRSA isolates (MRSA#1, MRSA#2, and MRSA#3) from navel swab, tracheal aspirate and biopsy sample, and *S. aureus* ATCC 29213 strain. Clinical isolates were obtained from the department of clinical microbiology, faculty of medicine, Ege University, İzmir. Bacteria were stored in brain-heart infusion broth (Merck KGaA, Darmstadt, Germany) with 10% glycerin at ­80 °C. Moxifloxacin (Bayer Türk Chemistry, İstanbul, Turkey), vancomycin (Mustafa Nevzat Pharmaceuticals, İstanbul, Turkey), ciprofloxacin, clindamycin, gentamicin (İ. E. Ulagay Pharmaceuticals, İstanbul, Turkey), diclofenac, naproxen, ibuprofen (Abdi İbrahim Pharmaceuticals, İstanbul, Turkey), acetylsalicylic acid and acetaminophen (Bayer Türk Chemistry) were provided by the pharmaceutical companies. Stock solutions of NSAIDs and antimicrobials were prepared in dimethylsulfoxide (DMSO) and distilled water, respectively, and filter-sterilization (0.2 μm) (Merck KGaA) was performed.

### 2.2. Disk diffusion test

Inhibition zone diameters of the drugs on bacteria were determined with the disk diffusion method according to the recommendations of the Clinical and Laboratory Standards Institute (CLSI) [19]. *Staphylococcus aureus* ATCC 25923 was used as a control strain. Bacteria were grown on Mueller–Hinton Agar (MHA) (Merck KGaA) at 37 °C for 24 h. Bacterial suspensions were prepared with physiological saline solution and cell densities were adjusted to 0.5 McFarland turbidity with densitometer (Den-1, Biosan SIA, Riga, Latvia). Bacterial suspensions were spread on MHA plates, and sterile empty discs (6 mm in diameter, Oxoid, UK) were placed on inoculated plates. Ten microliters of each of the drugs (100 μg/disk) were added onto the disks, and plates were incubated at 37 °C overnight. Cefoxitin antibiotic discs (30 µg, Oxoid Germany GmbH, Wesel, Germany) were used as a reference. Each sample was studied in triplicate, and quality control ranges were evaluated according to CLSI criteria. The diameters of inhibition zones were measured after the incubation period and mean values were reported.

### 2.3. Microdilution method

Minimum inhibitory concentration (MIC) values of the drugs were determined by microdilution method as suggested by CLSI [19]. *Staphylococcus aureus* ATCC 29213 was used as control strain. Bacterial suspensions were prepared with fresh colonies grown on MHA overnight. Suspensions were adjusted to 0.5 McFarland and diluted at a ratio of 1:100 (vol/vol). Fifty microliters of cation-adjusted Mueller­Hinton broth (Merck KGaA) were pipetted in each well of sterile 96-well plates. Fifty microliters of drug solutions were added to the first wells and serial dilutions of drugs were performed. Fifty microliter suspensions of bacteria were added to the wells, and plates were incubated at 37 °C for 24 h. Each drug was tested in triplicate; the lowest concentration that prevented microbial growth visually was determined as the MIC value, and quality control ranges were evaluated according to CLSI criteria.

### 2.4. MIC alteration experiments

Effects of NSAIDs (100 μg/μL) on MICs of the antimicrobials were performed using the microdilution method as mentioned above with minor modification. The drug solutions (40 μL) were added to the first wells, and serial dilutions were performed. Each drug solution (10 μL) was added to the wells at final concentrations of 100 μg/μL. All the experiments were done in triplicate, and mean values were calculated.

### 2.5. Real-time qRT-PCR

#### 2.5.1. Preparation of inoculum and drug treatment conditions

Bacterial suspensions (0.5 McFarland) were inoculated in Luria–Bertani (LB) broth (Merck KGaA) with sterile swabs and incubated with orbital shaking (Thermo Fisher, MaxQ 6000, Thermo Fisher Scientific Inc.,Waltham, MA, USA) (200 rpm) at 35°C for 4 h. Samples were transferred to sterile tubes and incubated with orbital shaking (200 rpm) with final concentrations of agents at 100 μg/μL and MICs for incubation periods of 4 and 16 h. All treatment procedures were carried out for three biological replicates. The optical density of bacterial cells was adjusted to equal levels.

#### 2.5.2. RNA isolation and cDNA synthesis

One milliliter of each bacterial suspension was centrifuged, and total RNA was isolated from pellets using GeneJET RNA Purification Kit (Thermo Fisher Scientific Inc.) according to manufacturer’s instructions. Total RNA concentrations were calculated by measuring absorbance at 260 nm (nanoVette, Beckman Coulter, Indianapolis, IN, USA). Two nanograms of each total RNA were used to synthesize cDNA using High Capacity cDNA Reverse Transcription Kit (Applied Biosystems, Foster City, CA, USA). The cDNA samples were diluted 1/50. All reactions were performed in triplicate.

#### 2.5.3. Real-time qRT-PCR

To determine the effects of NSAIDs on mRNA expression levels of *spa* , *sarA* , *agr* RNAIII, *sigB* , *mepA* , *mgrA,* and *srtA* genes, real-time qRT-PCR was performed using LightCycler 480 Instrument II (Roche, USA). Reactions were carried out in a 96-well plate using LightCycler 480 SYBR Green I Master kit (Roche Molecular Systems, Branchburg, NJ, USA). Primer sequences are given in Table 1. Housekeeping 16S rRNA gene was used as a control to normalize data. PCR was as follows: 5 min at 95 °C for initial denaturation, followed by 45 cycles of denaturation at 95 °C for 10 s, annealing at 43 °C for 10 s and elongation at 72 °C for 10 s before melting curve analysis. Threshold cycle (CT) values were calculated using the LC480 2 software program. All expression levels were normalized to that of internal 16S rRNA and given as target gene/16S rRNA. Data are presented as a fold change in gene expression in the presence of NSAIDs compared to control groups using delta delta CT method. All samples were analyzed in triplicate.

**Table 1:** Primers used for real time qRT-PCR study.

Primer	Sequence (5’-3’)	Amplicon (bp)	Reference
16S rRNA	F: TCGTGTCGTGAGATGTTG	188	[23]
	R: CTGCCCTTTGTATTGTCC		
spa	F: TATGCCTAACTTAAATGCTG	119	[22]
	R: TTGGAGCTTGAGAGTCATTA		
agr RNAIII	F: GGGATGGCTTAATAACTCATA	174	[22]
	R: GGAAGGAGTGATTTCAATGG		
sarA	F: CATCAGCGAAAACAAAGAGAAA	146	[23]
	R: TTCTTTCATCATGCTCATTACGTT		
srtA	F: CTTATCCTAGTGGCAGCATATTTGT	146	[39]
	R: TGAGGTTTAGCTTGCTGCTT		
mepA	F: GCGTTGGTGCAGGAACTTAT	151	[24]
	R: GCTGCGATTTGATCACTGAA		
sigB	F: TTTCACCTGAGCAAATTAACCA	145	[23]
	R: TCTTCGTGATGTGATTGTCCTT		
mgrA	F: CAATGCTCAAAGACAAGTTAATCG	122	[23]
	R: TCTTGACGTTTACAGGAGATTCA		

### 2.6. Protein isolation and immunoblotting analysis

#### 2.6.1. Preparation of inoculum and total protein isolation

Preparation of cell extracts was performed with some modification of previously published protocol [20]. One milliliter of each bacterial suspension was centrifuged at 5.000 × g at 10 °C. Pellets were suspended in 1 mL fresh buffer A (20 mMTris-HCl, pH 7.5, 150 mMNaCl, 5% glycerol, and 100 µg/mL lysostaphin), and the cells were incubated at 42 °C for 5 min. Sonication was performed for 10 s with 30% power (Bandelin Sonopuls, Bandelin Electronic, Berlin, Germany), and suspensions were centrifuged at 10000 × g for 45 min to remove cell debris.

#### 2.6.2. SDS-PAGE and immunoblotting

The concentration of total protein was determined with standard protein bovine serum albumin (BCA) (Sigma-Aldrich Chemie GmbH, Taufkirchen, Germany) using Pierce BCA Protein Assay Kit (Thermo Fisher Scientific, Rockford, IL, USA). Each sample was adjusted to equal concentrations, and one-dimensional denaturing sodium dodecyl sulfate polyacrylamide gel electrophoresis (SDS-PAGE) was performed with 10% polyacrylamide gels in a Bio-Rad Protean-II electrophoresis system. Total proteins were separated on 12% SDS-PAGE gel and transferred onto nitrocellulose membranes, membranes were reversibly stained with Ponceau S as a loading control. Membranes were washed three times for 10 min with TBS buffer (10 mMTris-HCl, 150 mMNaCl, pH 7.5) and incubated for 1 h in blocking buffer (5% BSA, 0.1% Tween 20 in TBS buffer). Membranes were washed three times for 10 min in TBS-Tween-Triton buffer (20 mMTris-HCl, 500 mMNaCl, 0.05% Tween 20, 0.2% Triton X-100, pH 7.5). Membranes were then incubated with primer antistaphylococcal protein A (antispA) antibody (Novus Dahle GmbH, Lingen, Germany) solutions (1:1000) at 4 °C overnight. Membranes were washed three times for 10 min in TBS-Tween-Triton buffer and incubated with secondary antibodies (Goat anti-,,,rabbit IgG (H+L) Secondary Antibody, HRP, Thermo Fisher Scientific, Rockford, IL, USA) (1:7500) for 1 h at room temperature. Membranes were washed three times for 10 min in TBS-Tween-Triton. Super Signal West Pico Chemiluminescent Substrate kit (Thermo Fisher Scientific, Fockford, IL, USA) was used for chemiluminescence detection according to the manufacturer’s recommendations, and the Fusion FX-7 program was used with the imaging device (Vilber Lourmat, Collégien, France). All samples were analyzed at least in triplicate and representative images of these triplicates are given in the figures.

### 2.7. Statistical analysis

Statistical significance between the means of two groups was evaluated using t-test and P ≤ 0.05 was considered significant. Statistical analyses were performed using the GraphPad Prism 5 program.

## 3. Results

### 3.1. Antimicrobial activity

Antimicrobial effects of the drugs were determined using disk diffusion and broth microdilution methods. Mean inhibition zone diameters of drugs for standard strain and clinical isolates are shown in Table 2. Inhibition zones were obtained with diclofenac and ibuprofen with the volume of 100 µg per disk for all studied bacteria in the disk diffusion test. Diclofenac was found to have the highest inhibition zone diameters. The results obtained for standard strain and clinical isolates are presented in Table 2. All the tested drugs were active against studied bacteria with MIC values from 195 µg/mL to 6250 µg/mL. Resistance profiles and the MICs of antibiotics show that MRSA#1 isolate was resistant to clindamycin (MIC: 20 µg/mL), MRSA#2 isolate was resistant to clindamycin (MIC: 20 µg/mL) and intermediate susceptible to ciprofloxacin (MIC: 1.25 µg/mL), MRSA#3 isolate was resistant to moxifloxacin (MIC: 2.5 µg/mL), ciprofloxacin (MIC: 80 µg/mL), clindamycin (MIC: 10 µg/mL) and gentamicin (MIC: 80 µg/mL).

**Table 2:** Mean inhibition zone diameters (mm) and MICs (µg/mL) of NSAIDs.

Strains	ASA		ACE		DIC		IBU		NAP	
	mm	µg/mL	mm	µg/mL	mm	µg/mL	mm	µg/mL	mm	µg/mL
MRSA#1	-	780	-	6250	14.3	390	7.3	780	-	780
MRSA#2	-	780	-	6250	14	390	7.3	780	-	780
MRSA#3	-	780	-	6250	15.7	195	7	780	-	780
ATCC	-	390	-	6250	17.3	195	7.7	780	-	780

ASA: acetylsalicylic acid, ACE: acetaminophen, DIC: diclofenac, IBU: ibuprofen, NAP: naproxen, (-): no inhibition zone.

Effects of the drugs (100 µg/mL) on susceptibility of isolates to several antibiotics were determined using the microdilution method with 25 combinations, and new MIC values of antibiotics in the presence of the drugs are demonstrated in Table 3. The presence of diclofenac resulted in a 4-fold (MRSA#1), 3.33-fold (MRSA#2) and 2-fold (MRSA#3) decrease in MICs of ciprofloxacin, 6.66-fold decrease in MICs of clindamycin and 6.66-fold (MRSA#1 and MRSA#2) and 5.6-fold (MRSA#3) decrease in MICs of gentamicin against three MRSA isolates. The presence of ibuprofen and naproxen resulted in a 6.66-fold decrease in MICs of gentamicin against MRSA#1. Ibuprofen and naproxen also reduced MICs of gentamicin 5.33-fold/8-fold in MRSA#2 and 4-fold / 4.8-fold in MRSA#3, respectively. MICs of gentamicin were reduced 4-fold in MRSA#1 and MRSA#2 in the presence of acetaminophen while MIC of gentamicin was reduced 2-fold in the presence of acetylsalicylic acid in MRSA#3.

**Table 3:** Effects of NSAIDs on the MICs of the antibiotics (fold-change decrease in the MICs of antibiotics, *: fold-change increase).

Drugs	Bacteria			
	MRSA#1	MRSA#2	MRSA#3	ATCC
MOX+ASA	-	-	-	1.25
MOX+ACE	-	-	-	1.25
MOX+DIC	1.33	1.66	-	1.25
MOX+IBU	-	-	-	1.25
MOX+NAP	-	-	1.66	1.25
CIP+ASA	-	-	-	-
CIP+ACE	-	1.66	-	1.66
CIP+DIC	4	3.33	2	4
CIP+IBU	-	-	-	*1.2
CIP+NAP	-	-	-	*1.2
VAN+ASA	-	1.33	1.33	-
VAN+ACE	-	-	-	-
VAN+DIC	2.33	-	-	1.66
VAN+IBU	1.33	-	1.33	-
VAN+NAP	2	-	2	2
CLI+ASA	-	-	-	-
CLI+ACE	1.66	1.66	1.66	-
CLI+DIC	6.66	6.66	6.66	2.33
CLI+IBU	-	3.33	-	-
CLI+NAP	-	2.66	-	-
GEN+ASA	1.33	1.66	2	1.25
GEN+ACE	4	4	1.6	1.25
GEN+DIC	6.66	6.66	5.6	2.5
GEN+IBU	6.66	5.33	4	3
GEN+NAP	6.66	8	4.8	2.5

MOX: moxifloxacin, CIP: ciprofloxacin, VAN: vancomycin, CLI: clindamycin, GEN: gentamicin, (-): no alteration, the data was the mean of three replicates.

### 3.2. Gene expression analysis by real-time qRT-PCR

Real-time qRT-PCR results are expressed as n-fold alteration in the expression of the genes ( *sarA* , *agr* RNAIII, *sigB* , *mgrA* , *spa* , *mepA,* and *srtA* ) versus 16S rRNA in the presence of drugs relative to without drugs. We observed that the addition of NSAIDs caused alteration in each gene expression levels at different rates. The addition of 100 μg/mL drugs to cultures of bacteria resulted in significant downregulation in the expression of three genes ( *sigB* , *mepA,* and *mgrA* ) in two isolates. For instance, *mepA* gene expression in MRSA#1 was downregulated 4.8-fold and 5.9-fold after 16 h incubation in the presence of 100 μg/mL acetylsalicylic acid and naproxen, respectively. Downregulation of *mepA* (12.7-fold), *sigB* (8.3-fold) and *mgrA* (8.4-fold) was also observed with 100 μg/mL ibuprofen with 4 h incubation in MRSA#2. In addition, 100 μg/mL diclofenac incubation for 4 h resulted in significant downregulation in the expression of *sigB* (3.1-fold) and *mgrA* (3.7-fold) in MRSA#2 although slight upregulation of *sarA* (1.8-fold), *sigB* (1.6-fold), *mepA* (2.4-fold), and *mgrA* (2.7-fold) was observed in MRSA#3 after 16 h incubation in the presence of 100 μg/mL diclofenac. 

The addition of drugs at MIC levels to the cultures of bacteria with 4 h and 16 h incubation resulted in significant alterations on the expression levels of the genes (Table 4). Our results revealed that *sigB, mepA, mgrA,* and *srtA* were downregulated in MRSA#1; and *spa, sigB,* and *srtA* were downregulated in MRSA#2, in response to growth with acetylsalicylic acid at MIC levels for 4 h and 16 h. In response to growth with acetaminophen at MIC levels for 4 h and 16 h, *sigB, mgrA,* and *srtA* genes were downregulated in MRSA#1; *spa, sigB,* and *srtA* were downregulated in MRSA#2, and only *mgrA* was downregulated in MRSA#3 in our analysis. The addition of diclofenac at MIC levels to the cultures for 4 h and 16 h resulted in a significant downregulation of *srtA* and *spa* in MRSA#1 and MRSA#2, respectively. Growth of MRSA#1 in the presence of ibuprofen at MIC for 4 h and 16 h downregulated the expression of *sigB, mepA, mgrA,* and *srtA* . In contrast to the downregulation of the genes in the presence of acetylsalicylic acid and acetaminophen, *spa, sigB, mgrA,* and *srtA* genes were upregulated following 16 h of growth in the presence of ibuprofen at MIC level in ATCC strain. In addition, *mepA* and *srtA* genes were also downregulated in MRSA#1; *spa* and *srtA* genes were downregulated in MRSA#2 in the presence of naproxen at MIC for 4 h and 16 h.

**Table 4:** Effects of the drugs at MIC on the expression of the genes significantly (P ≤ 0.05) after 4 h and 16 h incubation (fold-change downregulation and P values, *: fold-change upregulation).

Drugs and incubation time		Genes of MRSA#1							Genes of MRSA#2						
		spa	sarA	RNAIII	sigB	mepA	mgrA	srtA	spa	sarA	RNAIII	sigB	mepA	mgrA	srtA
4 h	ASA	15(˂0.001)	-	-	5.6(˂0.001)	6.5(0.028)	4.7(0.027)	6.6(0.001)	17.9(0.002)	6.4(0.036)	-	5.9(0.027)	-	-	8.4(0.023)
	ACE	27.7(˂0.001)	-	-	5.8(0.001)	-	4.6(0.034)	3.4(0.009)	21.4(0.002)	9.9(0.024)	-	11.8(0.016)	-	-	8.8(0.022)
	DIC	7.2(˂0.001)	-	-	2.2(0.003)	-	-	2.2(0.012)	2.1(0.015)	-	-	-/3.1(0.006)	-	-/3.7(0.014)	-
	IBU	28.9(˂0.001)	-	-	9(˂0.001)	12.3(0.022)	4.6(0.029)	9.1(0.001)	26.5(0.002)	5.1(0.031)	-	9/8.3(0.017/0.007)	-/12.7(0.012)	-/8.4(0.007)	20.3(0.017)
	NAP	23.8(˂0.001)	-	-	5(˂0.001)	6.2(0.031)	-	7.1(0.001)	6.1(0.006)	-	-	-	-	-	10.8(0.02)
16 h	ASA	-	5.4(0.007)	5.7(0.04)	7.1(0.025)	6.1/4.8(˂0.001/0.034)	6.6(0.034)	6.7(0.001)	8(0.004)	-	-	10.1(0.027)	16(0.039)	15.4(0.01)	13.8(0.004)
	ACE	-	11.8(0.004)	8.3(0.033)	12.9(0.02)	13(˂0.001)	13.3(0.026)	14.7(0.001)	12.7(0.003)	-	-	30.4(0.021)	25.9(0.036)	31.4(0.009)	24.3(0.003)
	DIC	-	-	-	-	-	-	1.7(0.026)	3.14(0.016)	-	-	8.9(0.029)	8.4(0.048)	10.3(0.012)	6.4(0.007)
	IBU	-	-	-	9(0.022)	9.2(˂0.001)	4.6(0.043)	7.2(0.001)	-	-	-	-	-	-	-
	NAP	-	-	-	-	2.5/5.9(0.003/0.031)	-	3.2(0.004)	3.2(0.013)	-	-	10.1(0.027)	-	13.3(0.011)	10.1(0.005)
Drugs and incubation time		Genes of MRSA#3							Genes of ATCC strain						
		spa	sarA	RNAIII	sigB	mepA	mgrA	srtA	spa	sarA	RNAIII	sigB	mepA	mgrA	srtA
4 h	ASA	-	-	-	-	-	-	-	-	-	-	-	-	-	-
	ACE	-	-	-	6(0.015)	3.7(0.005)	4.4(0.009)	4(0.027)	-	-	-	-	-	-	-
	DIC	-	-	-	-	-	-	-	-	-	-	-	-	-	-
	IBU	-	-	-	6.3(0.013)	3.2(0.007)	6.2(0.005)	6.4(0.015)	-	-	-	-	-	-	-
	NAP	-	-	-	11.8(0.01)	3.8(0.005)	5(0.006)	11.3(0.011)	-	-	-	-	-	-	-
16 h	ASA	-	-	-	-	-	1.9(0.01)	-	-	-	-	2.7(0.018)	3.7(0.004)	3.7(0.007)	-
	ACE	-	-	2.8(0.024)	-	-	2.6(0.003)	-	-	-	-	2.8(0.021)	6.4(˂0.001)	3.7(0.011)	2.9(0.042)
	DIC	-	-/*1.8(0.032)	-	-/*1.6(0.044)	-/*2.4(0.042)	-/*2.7(0.04)	-	-	-	-	-	-	-	-
	IBU	-	-	-	-	-	-	-	*5.4(0.047)	-	-	*23.1(0.031)	-	*24.4(0.03)	*27(0.032)
	NAP	-	-	-	-	-	-	-	-	-	-	-	2(0.009)	-	-

MIC: minimum inhibitory concentration, (-): no alteration, (bold): alteration with 100 µg/mL drugs, the data was the mean of three replicates.

### 3.3. Immunoblotting

**Figure 1:**
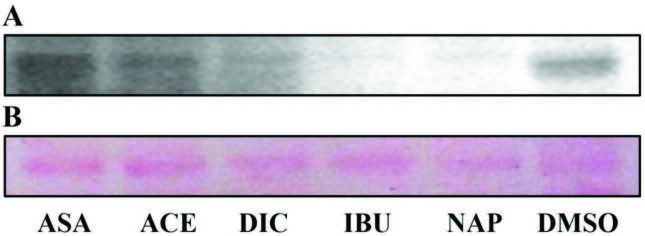
Western blot for detection of SpA of MRSA#1 isolate after treatment of the drugs at MICs for 4 h incubation (A). Ponceau S staining of the blot (B). DMSO: dimethyl sulfoxide. All samples were analyzed in triplicate and representative images of these triplicates are given.

**Figure 2:**
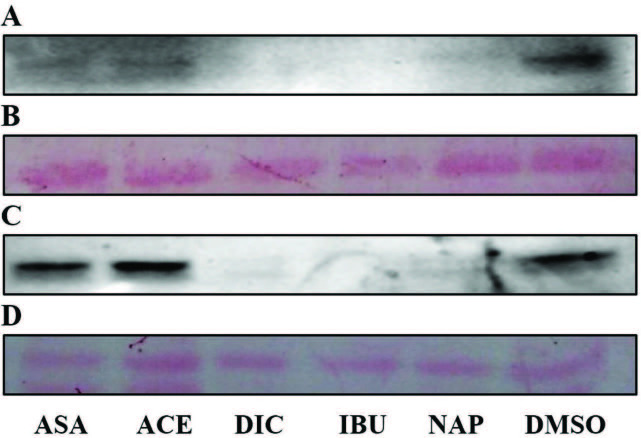
Western blots for detection of SpA of MRSA#2 isolate after treatment of the drugs at MICs for 4 h (A) and 16 h (C) incubation. Ponceau S staining of the blots (B and D). All samples were analyzed in triplicate and representative images of these triplicates are given.

*Staphylococcus aureus* has many virulence factors including toxins, cell wall-associated adhesins and secreted exoproteins, and these proteins can act in the process of several diseases. SpA protein, encoding by *spa* gene, represents an important role in interfering with host defence, and expression of SpA on the cell surface can cause bacteria less susceptible to host immune system. As shown in Table 5, in our experiments, we evaluated the effects of the drugs on SpA protein expression levels using immunoblotting, following drug treatment, total protein quantification, and SDS-PAGE. We examined samples where spa gene expression levels were found to be upregulated or downregulated in the presence of drugs. Immunoblotting analyses in SpA protein expression mostly correlates with the findings of significantly reduced transcriptional activity of the drugs (Table 5). In parallel, a decrease in SpA levels versus control group (DMSO group) was observed in two of four strains (MRSA#1: Figures 1A and 1B, MRSA#2: Figures 2A–2D).

**Table 5:** Alterations in the expression of spa gene and SpA protein after incubation for 4 h and 16 h in the presence of the drugs at MICs.

		MRSA#1		MRSA#2		MRSA#3		ATCC	
		spa	SpA	spa	SpA	spa	SpA	spa	SpA
ASA	4 h	↓	↓	↓	↓	-	nt	-	nt
	16 h	-	nt	↓	-	-	nt	-	nt
ACE	4 h	↓	↓	↓	↓	-	nt	-	nt
	16 h	-	nt	↓	-	-	nt	-	nt
DIC	4 h	↓	↓	↓	↓	-	nt	-	nt
	16 h	-	nt	↓	↓	-	nt	-	nt
IBU	4 h	↓	↓	↓	↓	-	nt	-	nt
	16 h	-	nt	↓	↓	-	nt	↑	↑
NAP	4 h	↓	↓	↓	↓	-	nt	-	nt
	16 h	-	nt	↓	↓	-	nt	-	nt

spa: staphylococcal protein A gene, SpA: staphylococcal protein A, (-): no alteration, (↓): downregulation, (↑): upregulation, nt: not tested.

**Figure 3:**
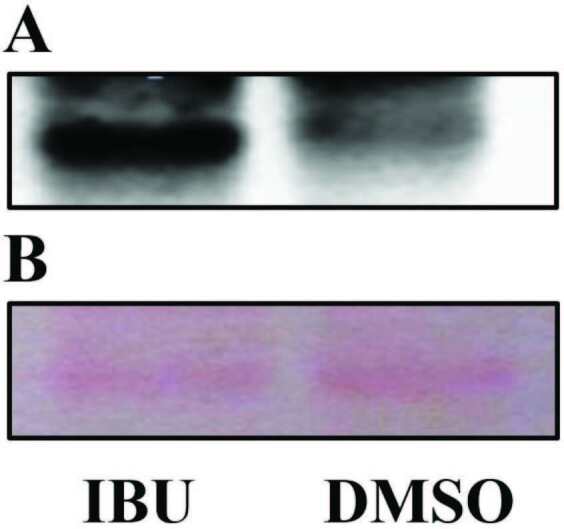
Western blot for detection of SpA of ATCC strain after treatment of ibuprofen at MIC for 16 h incubation (A). Ponceau S staining of the blot (B). All samples were analyzed in triplicate and representative images of these triplicates are given.

A strong decrease in SpA of MRSA#1 and MRSA#2 was determined in the presence of some drugs at MICs after 4 h incubation compared to control groups. Diclofenac, ibuprofen, and naproxen caused a strong decrease in SpA expression level of MRSA#2 at MIC after 4h and 16 h of incubation. Acetylsalicylic acid and acetaminophen had no inhibitory effect in the expression of SpA in MRSA#2 although expression of *spa* gene was decreased in the same treatment conditions. In contrast to these findings, an increase in SpA expression was determined in ibuprofen-treated cells of ATCC strain after 16 h incubation as compared with the untreated group (Figures 3A and 3B).

## 4. Discussion

In literature, it has been observed that the number of studies investigating the effects of NSAIDs and antimicrobials on various groups of bacterial virulence factors are limited [21–24]. In some studies, NSAIDs have been found to alter susceptibility of bacteria to antimicrobials [23,24]. Antimicrobial effects have been reported as most likely acting on multiple targets, and have been revealed by targeting DNA biosynthesis and replication, regulation of adhesins, toxins, biofilm and several metabolism pathways, and physiochemical effects on bacterial membrane [23–28]. In this study, we investigated antibacterial effects of frequently used NSAIDs, and alterations in antibiotic susceptibility were studied by phenotypic methods before the experiments investigating the gene and protein expression levels.

According to the results of phenotypic experiments including the disk diffusion test, our results indicate that inhibition zones were obtained with diclofenac and ibuprofen. Thus, we noticed that antimicrobial activity test methods including water-based media in the presence of the substances that are insoluble or less soluble in water, lower the potential antimicrobial effects because of solubility and diffusion issues. In a study, Obad et al. evaluated antimicrobial activity of ibuprofen as pure substance, salt, and lysine form in different formulations with disk diffusion tests and obtained inhibition zones for *S. aureus* ATCC strain with more than 62.5 and 250 µg of ibuprofen/disk and ibuprofen lysine/disk, respectively [29]. In another study, Laudy et al. investigated the effects of NSAIDs on bacterial susceptibility to antibiotics and modulation of bacterial efflux pumps and determined the activity of the drugs against clinical gram-negative bacteria by microdilution method [30]. Laudy et al. demonstrated that MICs of diclofenac, acetylsalicylic acid, ibuprofen, and paracetamol were between the range of 800 and 3200 µg/mL against clinical isolates. Al-Janabi reported that clinical *S. aureus* and *Paracoccus yeei* isolates were found to be susceptible to ibuprofen and acetaminophen at 1250 µg/mL concentration [31]. In addition, MIC values of diclofenac sodium were determined between the ranges of 50 and 100 µg/mL against various *S. aureus* strains in previous studies [25,32,33]. In this study, we observed inhibition zones with 100 µg/disk of ibuprofen and diclofenac against standard strain and three clinical MRSA isolates, and we observed low MIC values with the addition of NSAIDs against clinical gram-positive bacteria when compared to other results involving MICs against clinical strains.

The presence of NSAIDs mostly resulted in a decrease in MICs of antibiotics. In previous studies, it has been reported that active ingredients of salicylate and acetyl salicylate may be effective in the increase of resistance rates to fluoroquinolones, and despite the growth in the presence of salicylate may reduce MICs of some antibiotics [34,35]. Importantly, we demonstrated that acetylsalicylic acid slightly reduced MICs of gentamicin and moxifloxacin in both gentamicin susceptible and resistant *S. aureus* strains. Riordan et al. examined susceptibility of eight *S. aureus* strains to seven antibiotics in the presence of diclofenac using agar diffusion and drug gradient plate analysis [24]. They revealed that 32 and 64 μg/mL diclofenac increased susceptibility of *S. aureus* strains to quinolones (ciprofloxacin, norfloxacin, and ofloxacin) in a concentration and strain-dependent manner. Moreover, their results showed that diclofenac decreased susceptibility of *S. aureus* to oxacillin and vancomycin, but did not alter MICs for chloramphenicol or tetracycline. In this study, in parallel with findings of Riordan et al. [24], diclofenac resulted in 2-fold to 4-fold decrease in MICs of ciprofloxacin including MICs of ciprofloxacin in intermediate and resistant isolates. We also demonstrated that diclofenac decreased MICs of various antibiotics such as moxifloxacin, vancomycin, clindamycin, and gentamicin. Furthermore, we found that the presence of ibuprofen and naproxen resulted in a decrease in MICs of gentamicin against *S. aureus* strains. Additionally, we showed that MICs of gentamicin were reduced in two isolates in the presence of acetaminophen, and the MIC of gentamicin was reduced in one isolate in the presence of acetylsalicylic acid. Moreover, MICs of gentamicin were decreased in the presence of diclofenac, ibuprofen, and naproxen in resistant MRSA#3 isolate. Although it has been indicated that the growth in the presence of these NSAIDs can be both beneficial and detrimental, our results suggest that the presence of these drugs may result in a decrease in MICs of several antibiotics. Thus the effects of drugs on susceptibility to several antibiotics have been determined in detail, and the effects of drugs on transcriptome/protein A alterations described in *S. aureus* .

Global regulatory genes of staphylococci coordinate expression of various groups of genes, and expression rates may have clinical importance. Staphylococcal infections appear to require different combinations of virulence determinants in different stages of infections. Expression of surface proteins favors colonization of host tissues, whereas synthesis of exoproteins favors the spread to adjacent tissues [8]. Regulatory genes can alter the expression rates of many genes (such as enzymes, toxins, cell wall surface adhesives etc.) associated with virulence factors, either directly or inversely. Since several genes related to virulence factors are likely to be produced in very low amounts in bacteria, the detection of these genes at RNA and protein levels is more difficult than regulatory and structural genes, and regulatory genes were predominantly preferred in our experiments. In this study, the effects of the drugs differed among the agents, and our findings on transcriptional activity of drugs mostly correlate with findings of immunoblotting analyses. In addition, our results suggest that the alterations in the expression of certain genes altered expression rates of related genes in a similar way, and they are thought to interact with each other when compared with the downregulation rates.

The downregulation of *spa* gene was found to be parallel to SpA protein by the majority of our experiments. Although the downregulation in mRNA expression levels of *spa* gene in a clinical isolate were determined in the presence of drugs for 16 h, we were unable to detect an alteration in the expression of SpA protein in two acetylsalicylic acid-treated and acetaminophen-treated samples under our experimental conditions. Additionally, we demonstrated the downregulation of *spa* gene and SpA protein expression levels in two ibuprofen-treated clinical isolates. Interestingly, parallel to the upregulation rates of *spa* gene, an increase in SpA protein was determined in ibuprofen-treated cells of nonclinical ATCC strain after 16 h incubation compared with untreated group. We suggest that, in addition to the impact on *spa* gene, ibuprofen can mediate an impact on the upregulation and downregulation of different regulatory and virulence-related genes including *sigB* , *mgrA,* and *srtA* as shown in the results of our transcriptional experiments. Kupferwasser et al. also reported an increase of SpA protein and *spa* gene expression levels in salicylic acid-treated cells of *S. aureus* [36].

It is known that SpA has the ability to interact with the host components, including the immune system, and the expression of SpA on the bacterial cell surface can cause the bacteria to be less susceptible to phagocytosis. In this study, parallel to the findings on *spa* gene, we demonstrated that the expression of *srtA* gene was downregulated in the presence of NSAIDs, and the expression levels depended on the drugs and treatment time. In addition to the role of SpA, SrtA may have an important role in the virulence of *S. aureus* including the bacterial adhesion to host tissues and evasion of host immune components directly and/or indirectly. Newly discovered inhibitors of SpA and SrtA proteins may act as antimicrobials and may affect pathogenesis of bacterial infections with several mechanisms of action.

The *sigB* is a global stress regulon, it has been indicated in many studies that *sigB* negatively regulates the function of several adhesin genes and leads to repression of *sarA* and *agr* global regulons in *S. aureus* . Kupferwasser et al. investigated antivirulence properties of salicylic acid in *S.* *aureus* strains [36]. They demonstrated that *sarA* and *agr* were mitigated by salicylic acid in vitro, corresponding to the reduced expression of *hla* and *fnbA* genes. They also confirmed the key roles of *sarA* and *sigB* in vivo, in mediating antistaphylococcal effects in experimental endocarditis. Kupferwasser et al. reported a mean increase of SpA levels (17.6% ± 3.9%) in *S. aureus* strains exposed to 50 µg/mL salicylic acid [36]. They also reported a corresponding 2-fold increase in *spa* gene expression in salicylic acid-treated cells. In contrast, we demonstrated that *spa* gene expression was downregulated in two of four strains in response to growth with acetylsalicylic acid. In this study, we reported that the expression of *spa* gene was upregulated only in the presence of ibuprofen. Our immunoblotting analyses in SpA protein expression mostly correlate with the findings of transcriptional analyses. Interestingly, our results revealed that *sigB* , *sarA,* and *agr* RNAIII were downregulated in MRSA#1 in the presence of acetylsalicylic acid and acetaminophen; and *sigB* and *sarA* were downregulated in MRSA#2 in the presence of acetylsalicylic acid, acetaminophen, and ibuprofen at MICs. Our results suggest that, while *sigB* negatively regulates *sarA* and *agr* global regulons in low concentrations, the presence of studied drugs at high levels may eliminate the suppressive effect of *sigB* . 

MgrA (also known as Rat or NorR) is one of the SarA paralogs that are involved in the regulation of *sarA* family genes and virulence genes, and the SarA-SarR pathway may be involved in positive regulation of *agr* transcription in the exponential phase of growth [37]. It is also known that *mgrA* is involved in positive regulation of *agr* transcription in *S. aureus,* and a mutation in *mgrA* resulted in altered expression of *agr* RNAIII, *sarS* , *hla,* and *spa* genes [38]. Ingavale et al. reported that *mgrA* had a dual role in regulating *hla* and *spa* expression, and decreased *agr* transcription in *mgrA* mutants led to reduced *hla* transcription and an increase in *spa* transcription [38]. Riordan et al. investigated the transcriptome alterations and physiological responses that occurred in *S. aureus* cells exposed to 2 mM sodium salicylate [23]. They reported the downregulation of *mgrA* and *sarR* (repressor of *sarA* gene) and upregulation of antibiotic target genes such as *parE* and *fusA* [23]. In this study, we demonstrated that a decrease in *mgrA* transcription in the presence of acetylsalicylic acid and acetaminophen led to downregulation in the expression of *sarA* and *agr* RNAIII in isolate MRSA#1. We also reported that the downregulation of *mgrA* gene led to decreased expression of *spa* gene and SpA protein in the presence of acetylsalicylic acid, acetaminophen, and ibuprofen in MRSA#1, and in the presence of diclofenac and naproxen in MRSA#2. In this study, parallel to the findings of Riordan et al., *S. aureus* strains did not demonstrate increased resistance to ciprofloxacin and other antibiotics [23]. It is known that *mgrA* is a repressor of efflux pump genes, and we speculate that studied drugs can be effective in the decrease of MIC levels of fluoroquinolones and other group of antibiotics via *mgrA* -independent pathway. 

Transcriptome alterations in *S. aureus* strain were described in addition to the alterations in susceptibilities when grown with diclofenac [24]. Riordan et al. suggested that diclofenac altered the expression of regulatory/structural genes associated with cell wall biosynthesis/turnover and transport [24]. They reported that diclofenac induction led to the downregulation of *mepA* and *mepB* , revealing that the mepRAB operon was being repressed in the presence of diclofenac. It is known that *mepA* gene encodes MATE family efflux pump, and we demonstrated that the MIC of ciprofloxacin was reduced 2-fold in one isolate in the presence of 100 µg/mL diclofenac. Interestingly, *mepA* gene was upregulated in one isolate in the presence of 100 µg/mL diclofenac, although the expression rates were downregulated or not altered in the presence of several drugs. As a consequence, we also speculate that diclofenac can demonstrate increased susceptibility to ciprofloxacin by *mepA* -independent pathway.

In conclusion, our results indicate that studied NSAIDs may hold promise as new agents for preventing and reducing the severity of infections caused by *S. aureus* . Our findings demonstrate that the targeting of regulatory/virulence genes and protein A may be a reasonable strategy in the field of infection control and antimicrobial resistance. More experimentation will be required to investigate the understanding of the mechanism of gene/protein regulation in bacterial strains by NSAIDs.

## Informed consent

This article does not contain any studies involving animals and human participants performed by any of the authors.
